# Dynamic Responses of Microglia in Animal Models of Multiple Sclerosis

**DOI:** 10.3389/fncel.2020.00269

**Published:** 2020-08-20

**Authors:** Melanie J. Plastini, Haritha L. Desu, Roberta Brambilla

**Affiliations:** ^1^The Miami Project To Cure Paralysis, Department of Neurological Surgery, University of Miami Miller School of Medicine, Miami, FL, United States; ^2^The Neuroscience Program, University of Miami Miller School of Medicine, Miami, FL, United States; ^3^Department of Neurobiology Research, Institute of Molecular Medicine, University of Southern Denmark, Odense, Denmark; ^4^BRIDGE—Brain Research Inter-Disciplinary Guided Excellence, Department of Clinical Research, University of Southern Denmark, Odense, Denmark

**Keywords:** microglia, neuroinflammation, neurorepair, multiple sclerosis, neurological disease

## Abstract

Microglia play an essential role in maintaining central nervous system (CNS) homeostasis, as well as responding to injury and disease. Most neurological disorders feature microglial activation, a process whereby microglia undergo profound morphological and transcriptional changes aimed at containing CNS damage and promoting repair, but often resulting in overt inflammation that sustains and propagates the neurodegenerative process. This is especially evident in multiple sclerosis (MS), were microglial activation and microglia-driven neuroinflammation are considered key events in the onset, progression, and resolution of the disease. Our understanding of microglial functions in MS has widened exponentially in the last decade by way of new tools and markers to discriminate microglia from other myeloid populations. Consequently, the complex functional and phenotypical diversity of microglia can now be appreciated. This, in combination with a variety of animal models that mimic specific features and processes of MS, has contributed to filling the gap of knowledge in the cascade of events underlying MS pathophysiology. The purpose of this review is to present the most up to date knowledge of the dynamic responses of microglia in the commonly used animal models of MS, specifically the immune-mediated experimental autoimmune encephalomyelitis (EAE) model, and the chemically-induced cuprizone and lysolecithin models. Elucidating the spectrum of microglial functions in these models, from detrimental to protective, is essential to identify emerging targets for therapy and guide drug discovery efforts.

## Introduction

Microglia play an essential role in maintaining homeostasis in the central nervous system (CNS), as well as responding to injury and disease (Tay et al., 2017b). Most neurological disorders feature microglial activation, a process whereby microglia undergo profound morphological and transcriptional changes aimed at containing CNS damage and promoting repair. However, prolonged and dysregulated microglia activation may result in damaging inflammation that sustains and propagates the neurodegenerative process. This is especially evident in multiple sclerosis (MS), a chronic demyelinating CNS disorder whose initiation, progression, and clinical course are dictated by a combination of dysregulated immunity, genetic predisposition, and environmental factors (Thompson et al., 2018). Microglia are prominent in MS immunopathology and take on specific roles depending on anatomical location and disease phase. Microglial activation and microglia-driven neuroinflammation have been recognized as key events in the onset, progression, and resolution of MS (Voet et al., 2019).

Our understanding of microglial functions in MS has expanded in the last decade by way of new tools and markers to discriminate microglia from other myeloid populations (Ginhoux et al., 2010; Goldmann et al., 2013). Consequently, the complex functional and phenotypical diversity of microglia can now be appreciated (Masuda et al., 2020). This, in combination with a variety of animal models that mimic specific features and processes of MS pathophysiology, has helped fill the gap of knowledge in the cascade of events that sustain initiation, progression, and resolution of the disease.

The purpose of this review is to present the most up to date knowledge of the dynamic responses of microglia in the commonly used animal models of MS, specifically the immune-mediated experimental autoimmune encephalomyelitis (EAE) model, and the chemically-induced cuprizone and lysolecithin models. Elucidating the spectrum of microglial functions in these models, from detrimental to protective, is essential to identify emerging targets for therapy and guide drug discovery efforts.

## Microglia in Health and Disease

Known as the resident immune cells of the CNS, microglia are a highly specialized population of mononuclear phagocytes whose origin has been traced to yolk sac progenitors colonizing the neuroepithelium during developmental hematopoiesis (Ginhoux et al., 2010; Gomez Perdiguero et al., 2015). Once established in the CNS, microglia can repeatedly self-renew over the individual’s life span through coordinated apoptotic and proliferative processes (Askew et al., 2017). Self-renewal occurs randomly in homeostatic conditions but becomes targeted in disease states with site-specific clonal expansion of select microglia clusters to respond to local perturbations (Tay et al., 2017a).

In the adult CNS, the primary role of microglia is to preside over tissue homeostasis and carry out surveillance functions to prevent any disturbances to CNS integrity. Their highly ramified and plastic morphology, as well as their motility, allow them to reach into the microenvironment and sense alterations caused by endogenous and exogenous signals. This capability is attributed to a molecular machinery unique to microglia encoded by a cluster of genes that have been collectively defined as the microglial sensome (Hickman et al., 2013).

The distribution, number, and phenotype of microglial cells is condition and region-dependent, with the white matter (WM) containing more microglia than the gray matter (GM; Mittelbronn et al., 2001). Genome-wide transcriptional profiling of microglia from various areas of the adult mouse brain has shown that, aside from the defined core of sensome genes, microglia possess region-specific transcriptional profiles that account for a high degree of cellular heterogeneity. This has also been shown by *ex vivo* flow cytometric analysis, whereby microglia has been found to have region-specific differences in the expression of immunoregulatory proteins (de Haas et al., 2008). The diversity of their molecular repertoire enables microglia specialized homeostatic functions and may explain their varying responses in CNS pathological states (Grabert et al., 2016).

Phagocytosis is a key function of microglia. During development, particularly in the early postnatal period, microglia deploy their phagocytic capability to remove excess neurons and synapses, shaping the structure of adult neuronal networks (Paolicelli et al., 2011; Schafer et al., 2012). This function is also executed through the production of trophic and synaptogenic factors (Parkhurst et al., 2013; Ueno et al., 2013). In pathological conditions, phagocytic microglia clear cellular debris and pathogens, making way for reparative processes (Galloway et al., 2019b).

In response to injury and disease, microglia undergo a process of cellular activation characterized by morphological changes (e.g., amoeboid, enlarged, sphere-like cell body with shorter branching), increased cell proliferation, and functional modifications that include the production of soluble mediators. These processes can result in both detrimental and protective effects depending on timing and location and together influence the neurological outcome. Moving away from the classification that labeled damaging microglia as M1 and reparative microglia as M2, single-cell transcriptomic studies have unequivocally established that, *in vivo*, microglia exist in a multitude of dynamic states constantly interchanging (Martinez and Gordon, 2014; Kim et al., 2016; Masuda et al., 2019, 2020). As effectors of the innate immune response, microglia express a variety of chemotactic mediators that sustain the trafficking and activation of immune cells recruited at the site of damage. They also acquire antigen presentation capability by expressing MHCII and co-stimulatory molecules such as CD40 (Butovsky and Weiner, 2018).

## Microglial Responses in Multiple Sclerosis

Multiple sclerosis is a chronic inflammatory disease of the CNS, whose onset and progression have been attributed to the interplay of aberrant immune system activation (both innate and adaptive), genetic susceptibility, and environmental factors (Dendrou et al., 2015). Its clinical hallmarks range from sensory, visual, and motor disturbances to cognitive dysfunction and fatigue (Compston and Coles, 2008). MS manifests with distinct phenotypes, all characterized, to various extents, by compromised blood-brain barrier (BBB) permeability, infiltration of immune cells into the CNS parenchyma, and glial activation (Dendrou et al., 2015; Brambilla, 2019). Together, these events synergize to induce and propagate neuroinflammation, the formation of demyelinating lesions, and ultimately neurodegeneration (Dendrou et al., 2015).

Microglial activation is a prominent feature in all stages and forms of MS. Indeed, histological characterization of microglial morphology and the expression pattern of select markers in normal and pathological conditions has allowed a detailed classification of MS lesions in relation with disease stage and evolution (van der Valk and De Groot, 2000; Zrzavy et al., 2017). In the normal brain, microglia display low expression of CD68, CD45 and HLA-DR (MHC class II receptor) molecules, and high expression of homeostatic markers such as the purinergic receptor P2RY12 (van der Valk and De Groot, 2000; Zrzavy et al., 2017). In the early stages of lesion development, microglia form clusters, or nodules, within the normal-appearing white matter (NAWM) with no signs of demyelination. These structures, classified as *pre-active* or *early active lesions*, are uniquely composed of microglia with upregulated CD68, CD45, HLA-DR, and are not accompanied by BBB alterations nor astrogliosis, but are associated with degenerating axons. Here, microglia show both pro-inflammatory and pro-reparative (e.g., expression of TNF, NADPH oxidase-2 subunits, and IL10) signatures (Howell et al., 2010; van Horssen et al., 2012; Singh et al., 2013). In *active WM lesions*, microglial activation increases with further upregulation of CD68, CD45, HLA-DR, and B7 costimulatory molecule (De Simone et al., 1995), and microglial processes are observed in close contact with transected axons (Trapp et al., 1998). At this stage, microglia lose their homeostatic signature, for example downregulating expression of P2RY12 in favor of the inflammatory P2X7 receptor (Beaino et al., 2017; Zrzavy et al., 2017). *Chronic active WM lesions* are characterized by a hypocellular demyelinated center surrounded by a rim of CD68^+^ microglia/macrophages containing residual lipids. *Chronic inactive demyelinated WM lesions* are also hypocellular, with rare residual CD68^+^ microglia/macrophages (Kuhlmann et al., 2017).

A similar classification based on microglia morphology has been established for gray matter (GM) lesions, where microglial activation has been associated with cortical demyelination and neurodegeneration (Trapp et al., 1998; Magliozzi et al., 2010, 2013; Reynolds et al., 2011). Here, microglial activation follows a gradient pattern, higher in the superficial layers close to the meningeal surface where GM damage is most severe, and progressively lower in the deepest layers of the cortex (Magliozzi et al., 2010).

Histological studies on post-mortem MS tissue revealed that microglia in active and chronic active MS lesions produce a variety of molecules, which have been attributed both detrimental and neuroprotective functions (Voet et al., 2019). These include cytokines such as TNF and TNF family members (e.g., lymphotoxin, TWEAK), but also IL1β, IL6, IL12, IL23, and IL33, all of which have been mostly associated with damaging inflammatory processes (Selmaj et al., 1991; Li et al., 2007; Serafini et al., 2008; Christophi et al., 2012). In a recent study by Magliozzi et al. (2019), loss of TNFR2-mediated protective TNF signaling in microglia in favor of enhanced detrimental TNFR1 signaling in neurons and oligodendrocytes has been directly implicated in the development and severity of submeningeal GM lesions.

Chemokines (e.g., CCL4, CCL5, CCL8, CXCL9, CXCL10, CXCL2, and CXCL4), which play a role in the recruitment of T cells and monocytes into the CNS, are also produced by microglia (Selmaj et al., 1991; Li et al., 2007; Serafini et al., 2008; Saikali et al., 2010; Christophi et al., 2012). Elevated expression of the chemokine receptors CCR5, CCR8 and CXCR4 has been described in microglia at lesion sites (Trebst et al., 2001, 2003, 2008; Moll et al., 2009), indicating they respond to chemoattractant cues to reach the demyelinating lesion environment. Interestingly, CCR5^+^ microglia with phagocytic morphology are found not only in active lesions but also in early remyelinating lesions, suggesting that this microglia population may be associated with damaging phagocytic activity at the acute stage of lesion activity and reparative phagocytosis to clear debris at a later stage to favor the remyelination process (Trebst et al., 2008).

The introduction of single nucleus transcriptomics on post-mortem MS tissue has marked a turning point in our appreciation of the functional diversity of microglia in the MS affected CNS. Within and close to lesion areas of the WM and GM, microglia lose their homeostatic signature and transition into a variety of activated phenotypes (e.g., upregulation of CD163, CD68, CD74, FTL, MSR1) that dynamically change as the disease evolves (Schirmer et al., 2019). Microglia show different gene signatures in GM and WM. In the GM, microglia upregulates the expression of glycolysis and iron homeostasis genes, whereas in the WM lipid metabolism genes are increased, demonstrating region-specific functional roles for microglia (van der Poel et al., 2019).

Activated microglia has also been shown to participate in tissue damage caused by reactive oxygen species (ROS) in MS. Indeed, ROS producing enzymes such as myeloperoxidase (MPO) and nicotinamide adenine dinucleotide phosphate (NADPH) oxidase subunits have been found upregulated in microglia within and in the proximity of MS lesions, both in WM and GM (Nagra et al., 1997; Gray et al., 2008a,b; Fischer et al., 2012).

## Experimental Autoimmune Encephalomyelitis (EAE)

**Figure 1:**
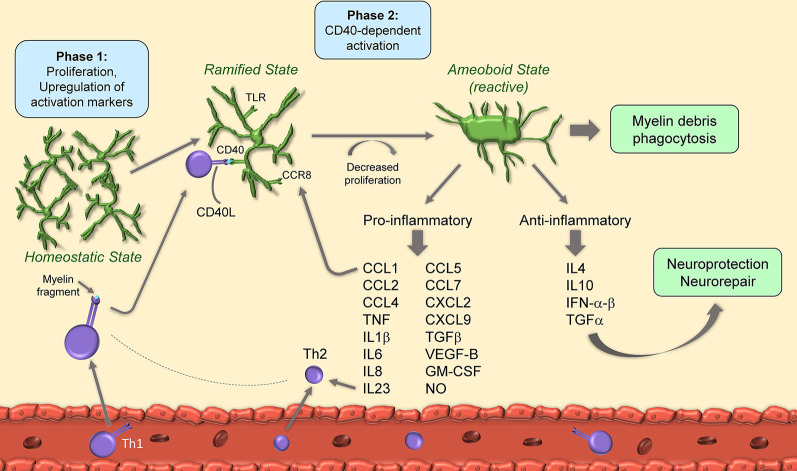
Schematic of microglia activation and responses during experimental autoimmune encephalomyelitis (EAE). After EAE induction, microglia begin to proliferate and upregulate activation markers. Upon binding of CD40L from T cells to microglial CD40, microglia decrease their proliferation rate and acquire amoeboid reactive morphology. Amoeboid microglia have both pro- and anti-inflammatory functions through secretion of cytokines, chemokines and growth factors.

EAE is the most utilized model of MS (Constantinescu et al., 2011). Typically, it is induced *via* immunization with synthetic peptides matching highly immunogenic regions of myelin proteins such as myelin oligodendrocyte glycoprotein (MOG), myelin basic protein (MBP), and proteolipid protein (PLP). Peptides are injected emulsified in Complete Freund’s Adjuvant (CFA) to boost immune activation and are usually accompanied by administration of pertussis toxin, which is thought to favor BBB breakdown and facilitate immune cell extravasation into the CNS parenchyma. Being T cell-mediated, EAE is especially suited to mimic the pathological hallmarks of the acute and relapsing-remitting phases of MS and has paved the way for the development of first-line disease-modifying therapeutics currently in clinical use. Similar to MS, EAE features profound immune-inflammatory activation sustained by the synergistic action of immune cells trafficking into the CNS and resident glia, especially microglia (Figure 1).

### Detrimental Microglial Activation in EAE

As with MS, we are beginning to appreciate the diversity and complexity of the microglia repertoire in response to the EAE challenge. By single-cell RNAseq (scRNAseq) analysis, at least four disease-associated microglia subsets were identified following EAE, three of which showed high upregulation of inflammatory and proliferative genes (Ly86, Mki67, CCL2, CCL5, and CXCL10) and were localized to demyelinating lesions (Jordão et al., 2019).

It has been suggested that microglial activation is a two-phase process in EAE. The first, occurring at the onset, is independent of CD40, a costimulatory molecule found on antigen-presenting cells and required for their activation, and also known to augment CD3 mediated T cell activation (Munroe and Bishop, 2007): this phase features cell proliferation and upregulation of the activation markers MHC-II, CD40 and CD86. The second, at disease peak, is CD40-dependent, features further upregulation of activation markers, and is paralleled by a reduction in cell proliferation. At this stage, CD40-dependent microglial activation is necessary for encephalitogenic T cell expansion and for the continued infiltration of leukocytes which sustain chronic disease progression (Ponomarev et al., 2006). Importantly, T cells themselves produce CD40 ligand (CD40L), hence directly influence microglial reactivity by binding to microglial CD40 in a positive feedback loop (Ponomarev et al., 2006). Studies indicate that microglial control of T cell encephalogenicity occurs through IL23, specifically *via* the p40 subunit. Without microglial p40, EAE is suppressed due to a shift towards a Th2 rather than Th1 phenotype (Becher et al., 2003).

Some of the intracellular signals that control microglial activation in EAE have been identified thanks in part to the development of CX3CR1-Cre^ER^ mice that allow for microglia-specific conditional gene knockout. CX3CR1 itself, primarily expressed by microglia in the CNS, is crucial for mediating cellular activation. Its ablation leads to increased microglial activation which parallels an early EAE onset and a more severe clinical course (Cardona et al., 2006; Wlodarczyk et al., 2015). Another important signal for microglia activation is the TGFβ-activated kinase 1 (TAK1), as its ablation prevents microglia from acquiring activated amoeboid morphology and producing pro-inflammatory IL1β and CCL2. This leads to suppressed EAE with reduced immune cell infiltration and demyelination (Goldmann et al., 2013). The NF-κB regulatory protein A20 also plays a role in microglial activation following EAE. Its deletion causes hyperactivation of the NLRP3 inflammasome with enhanced IL1β release and exacerbated neuroinflammation (Voet et al., 2018).

As part of the activation process, microglia ramp up production of inflammatory mediators, which include cytokines (e.g., TNF, IL1β, IL6) chemokines (e.g., CCL1, CCL2, CCL5, CCL7, CXCL2), complement factors (e.g., C4a) and nitric oxide (NO; Renno et al., 1995; Villarroya et al., 1996; Tran et al., 1997; Lewis et al., 2014; Yamasaki et al., 2014; Stoolman et al., 2018). This is driven, at least in part, by IFNγ and IL17 secreted by T cells infiltrated in the CNS (Renno et al., 1995; Murphy et al., 2010). Microglial IL6 has a pathogenic role in EAE, as its conditional ablation significantly ameliorates EAE symptoms and reduces immune cell infiltration and demyelination (Sanchis et al., 2020). This may be due, in part, to stimulation of IL6 receptors (IL6R) on endothelial cells, causing BBB disruption and increased CNS immune cell trafficking (Petković et al., 2020). Similarly, IL1β and IL18 produced by microglia are neurotoxic, and preventing their production *via* administration of the caspase-1 inhibitor VX-765, which blocks the inflammasome pathway, is therapeutic in EAE (McKenzie et al., 2018). Along the same line, microglia derived growth factors sustain detrimental CNS inflammation in EAE. Transforming growth-factor-β (TGFβ) is produced by microglia after activation of the Angiotensin II type-1 receptor (AT1R) by Angiotensin II (Ang II). Blockade of AT1R with candesartan, an anti-hypertensive drug, inhibited TGFβ production and improved EAE, suggesting this category of molecules may have a therapeutic effect in MS (Lanz et al., 2010). It should be noted that some studies suggest that TGFβ has beneficial functions in EAE (Lee et al., 2017). However, the protective functions of this cytokine during EAE have not been assessed in a cell-specific manner, thus it has not been determined whether or not they are dependent on microglia (Xu et al., 2019). Microglial VEGF-B triggers FLT-1 signaling in astrocytes causing activation of pro-inflammatory NF-κB signaling, upregulation of NF-κB-dependent cytokines, and worsening of EAE (Rothhammer et al., 2018).

In addition to cytokines, diverse signals have been shown to promote detrimental microglial activation in EAE, such as the CCR8-CCL1 axis. Indeed, CCL1, highly produced by microglia after EAE, by interacting with its cognate receptor CCR8, also expressed in microglia, sustains cell activation at EAE onset (Murphy et al., 2002). This mechanism has been suggested to take place in MS as well (Trebst et al., 2003). Stimulation of toll-like receptor (TLR) signaling leads to microglia activation and results in the production of soluble mediators, including TNF, IL10, IL6, CCL2, CCL5, and GM-CSF (Olson and Miller, 2004). Among the various TLRs, TLR2 is directly activated by 15-alpha-hydroxicholestene (15-HC), an oxidized cholesterol derivative found in the serum of patients with secondary progressive MS and mice with EAE. TLR2 activation by 15-HC caused detrimental neuroinflammation and correlated with increased microglial production of CCL2, iNOS, and TNF, as well as worsening of EAE symptoms (Farez et al., 2009). TLR signaling was shown to be regulated by the E3 ubiquitin ligase Peli1 *via* TRAF3 degradation, as ablation of Peli1 abrogated microglial activation and suppressed EAE (Xiao et al., 2013).

Microglial activation in EAE has been linked to direct impairment of neuronal function through various mechanisms (Mandolesi et al., 2015). Inflammatory mediators produced by microglia, especially TNF, have been shown to mimic the synaptic alterations in hippocampal glutamatergic signaling observed in EAE (Centonze et al., 2009). Also, ROS released by microglia *via* the activity of mitochondrial NADPH oxidase was associated with synaptic and cognitive alterations after EAE (Di Filippo et al., 2016). In the cerebellum, microglia produced IL1β was linked to altered glutamate transmission at Purkinje cell synapses following EAE (Mandolesi et al., 2013). In the cortex, contact of activated microglia with the axon initial segment responsible for action potential initiation caused axonal pathology independently of T cell presence, and this could be reversed by pharmacological deactivation of microglia with the ribonucleotide reductase inhibitor didox (Clark et al., 2016). Inhibition of mixed lineage kinases, which have been associated with microglial activation and neurodegeneration, reduced loss of postsynaptic structures (Bellizzi et al., 2018). Importantly, disease-modifying drugs used in MS therapy have shown efficacy in rescuing synaptic dysfunction in EAE through inhibition of detrimental microglial activation. These include fumarates, through blockade of NF-κB signaling (Parodi et al., 2015), and sphingosine-1-phosphate (S1P) receptor modulators such as fingolimod, laquinimod, and ozanimod, through reduction of the release of proinflammatory mediators (e.g., TNF) from microglia (Rossi et al., 2012; Gentile et al., 2018; Musella et al., 2020).

Overall, these studies indicate that persistent and overt microglial activation has a net detrimental role in CNS autoimmunity, and preventing or suppressing this process may be therapeutic. This has been demonstrated in a seminal study by Heppner et al., who provided the first direct evidence that “microglial paralysis”—intended as microglia with reduced capacity to proliferate, migrate and produce cytokines—leads to EAE suppression (Heppner et al., 2005). Indeed, *tg*620^chi^ transgenic mice with selective deactivation of microglia after administration of ganciclovir showed a marked delay in EAE onset as well as a reduction of the clinical disease score. This was associated with the absence of Iba1^+^ microglia and a lack of inflammatory infiltrates in the CNS (Heppner et al., 2005). Interestingly, it has also been shown that ganciclovir *per se* has a direct inhibitory effect on microglial proliferation and activation, suggesting that its beneficial effects in the CNS go beyond its known antiviral properties (Ding et al., 2014). Along the same line, induction of microglia apoptosis *via* administration of the anti-hypertensive drug nimodipine has proven beneficial in EAE. The apoptotic effect of nimodipine on microglia, independent of its calcium channel blocking effect, led to reduced NO and ROS and promoted remyelination (Schampel et al., 2017).

Colony-stimulating factor 1 receptor (CSF1R) is essential for microglial survival and proliferation. Microglia depletion induced pharmacologically by inhibition of CSF1R in a mouse model of EAE reduced neuroinflammation and increased myelin preservation, suggesting that the presence of microglia contributes to an environment that prevents remyelination and CNS recovery (Nissen et al., 2018). Similarly, in a rat model of EAE, it was found that administering the CSF1R inhibitor GW2580 slowed disease progression and reduced the EAE clinical scores (Borjini et al., 2016).

Further indication that keeping microglia reactivity in check is desirable, exogenous administration of miR-124, which inhibits the transcription factor C/EBP-α and its downstream targets polarizing microglia towards a quiescent phenotype, prevents EAE development (Ponomarev et al., 2011). Similarly, the administration of miR-146a, which inhibits microglial inflammatory activation by suppressing TLR2 signaling, inhibits EAE (Zhang et al., 2019). Notably, miR-146a is upregulated in EAE and MS (Fenoglio et al., 2011; Madsen et al., 2016), possibly indicative of an endogenous anti-inflammatory response that alone, however, is not sufficient to suppress disease. Astrocyte-derived Gal-1, by binding to core 2-O-glycans on CD45, is retained at the microglial cell surface, where it exerts a deactivating function by augmenting its phosphatase activity (Starossom et al., 2012). Estrogens, *via* stimulation of microglial ERβ, also promote microglia deactivation, and this occurs through inhibition of NF-κB signaling and downregulation of NO synthase (Wu et al., 2013). ERβ agonists have been proven effective in suppressing EAE (Wu et al., 2013; Moore et al., 2014).

### Protective Microglial Activation in EAE

Despite the large body of evidence pointing at a primary detrimental role of microglia in EAE, various reports depict a more complex picture of microglia involvement in EAE pathogenesis. It has been shown that the recruitment of peripheral monocytes/macrophages to the CNS is necessary for EAE onset and that activation of microglia alone is not sufficient (Ajami et al., 2011). This was also underscored in a study by Yamasaki et al. (2014) who identified differences in the gene expression profile of infiltrating macrophages and resident microglia at EAE onset. While macrophages are highly phagocytic and inflammatory driving disease, microglia demonstrate a signature of globally suppressed cellular metabolism, suggesting a lesser pathogenic role at EAE onset.

In response to signals present in the CNS environment at the various stages of EAE, microglia is directed towards producing beneficial factors and performing reparative functions to restore CNS homeostasis. One of these signals is IFNγ released by encephalitogenic T cells, as lack of microglial IFNγR resulted in exacerbated EAE through increased microglia proliferation (Ding et al., 2015). Microglia is also the major CNS source of type I interferons following EAE, including IFNβ, which, administered exogenously, is a standard treatment for relapsing-remitting MS (RRMS). Microglia expressing IFNα−β are localized within active demyelinating lesions and are highly efficient in clearing myelin debris through an enhanced phagocytic capacity (Kocur et al., 2015). Microglia not only produce type I interferons but are also responsive to them as they express IFNAR1. Stimulation of IFNAR1 leads to the expression of IFN-dependent genes including IFNα−β themselves, further sustaining the protective signaling evoked by this class of molecules. Although the amount of microglial type I interferons naturally produced during EAE is not sufficient to suppress EAE, stimulation with polyI:C, which upregulates IFNα-β in microglia, limits EAE development, suggesting this is a potent protective mechanism that microglia controls (Khorooshi et al., 2015).

During EAE, microglia upregulates the production of IL4, a cytokine known for its anti-inflammatory function. Mice lacking IL4 in the CNS are more susceptible to EAE, indicating that microglial IL4 serves as a suppressive signal that may balance overt pro-inflammatory cascades (Ponomarev et al., 2007). In parallel, microglia exposed *in vitro* to IL4 produce reparative, anti-inflammatory factors and could potentially be exploited for therapeutic purposes. Indeed, IL4-treated microglia delivered *via* adoptive transfer to EAE-induced mice reduced EAE severity and overall demyelination (Zhang X. M. et al., 2014). Microglia produced TGFα is also protective in EAE by limiting the pathogenic functions of astrocytes *via* activation of ErbB1 receptors and inhibition of NF-κB signaling (Rothhammer et al., 2018).

Interleukin-1 receptor-associated kinase (IRAK)-M is selectively expressed by microglia in the CNS and has been associated with shifting microglia towards an anti-inflammatory phenotype by inhibiting TLR4 signaling (Liu et al., 2019). Indeed, the ablation of IRAK-M in mice exacerbated EAE and increased pro-inflammatory microglia (Liu et al., 2019), suggesting that enhancing IRAK-M signaling could be a therapeutic option for MS.

Although some reports have shown that inhibition of CSF1R is protective in EAE (Borjini et al., 2016; Nissen et al., 2018), others suggest the opposite. Indeed, stimulation of CSF1R with its ligands CSF1 or IL34 increased protective CD11c^+^ microglia and ameliorated EAE symptoms (Wlodarczyk et al., 2018). Furthermore, depletion of microglia with a CSF1R antagonist exacerbated EAE and increased neurodegeneration and inflammation in the Non-Obese Diabetic (NOD) mouse strain EAE model of secondary progressive MS, suggesting that microglial CSF1R signaling may be protective in certain conditions (Tanabe et al., 2019). It should be noted that the NOD-EAE model of MS has a clinical course and pathological hallmarks different form the typical EAE models in the C57Bl/6 and SJL mouse strains. This could account for the lack of efficacy of CSF1R inhibition in this model.

An important pathway controlling the protective host-defense and homeostatic functions of microglia during EAE is the tumor necrosis factor receptor 2 (TNFR2) signaling pathway. Microglia-targeted ablation of TNFR2, which is activated by the transmembrane form of TNF, resulted in the early onset of EAE with exacerbated demyelination (Gao et al., 2017). TNFR2 deficient microglia showed enhanced pro-inflammatory profile while exhibiting deficiencies in homeostatic genes (e.g., P2X4R, P2X7R, P2Y12R, TREM2, and Siglech) and reduced phagocytic capacity. Microglial TREM2 is especially important in EAE repair, as TREM2 inhibition caused disease exacerbation with increased immune cell infiltration and demyelination (Piccio et al., 2007). Similar to TNFR2, microglial P2X4R is also important for host defense function. Its blockade exacerbated EAE by favoring pro-inflammatory microglia activation and inhibiting myelin phagocytosis (Zabala et al., 2018). On the contrary, potentiation of P2X4R signaling ameliorated EAE, promoted anti-inflammatory microglia activation, and potentiated myelin phagocytosis and remyelination (Zabala et al., 2018).

Collectively, these studies indicate that microglia are tasked with important homeostatic and reparative functions during EAE and their maintenance is crucial for repair and recovery.

## Cuprizone-Induced Demyelination

Administration of the copper chelator cuprizone, typically incorporated into the chow and fed to mice for 4–6 weeks, induces death of mature oligodendrocytes and consequent demyelination in the CNS, particularly in the corpus callosum (Matsushima and Morell, 2001). The underlying mechanism of this process or why myelinating oligodendrocytes are especially susceptible is not entirely clear. Evidence indicates that disruption of mitochondrial function and metabolism in oligodendrocytes may be implicated, particularly in the early days of cuprizone administration (Praet et al., 2014). However, at later stages of cuprizone administration, when the peak of oligodendrocyte death occurs, the activation of the innate immune response, especially of microglia, seems to be the key mechanism driving oligodendrocyte death (Praet et al., 2014). Notably, unlike the EAE model, demyelination in the cuprizone model is not dependent on nor accompanied by a T cell-mediated immune response, allowing for the study of mechanisms of demyelination/remyelination without confounding superimposed inflammatory mechanisms. Importantly, oligodendrocyte precursor cells (OPCs) are not susceptible to cuprizone-induced cell death. They proliferate and populate demyelination sites initiating the remyelination process, thus making the cuprizone model a reversible model of demyelination.

With cuprizone administration, evidence of microgliosis is observed within 1 week. Microglia numbers continue to rise through weeks 3–4 of cuprizone exposure, after which they plateau (Hiremath et al., 1998). Spontaneous remyelination has been shown to occur between weeks 5 and 6 of cuprizone administration due to OPC migration and differentiation at the sites of demyelination. This correlates with the time when microglia numbers reach their highest, suggesting that at this stage microglia may be important for the remyelination process (Mason et al., 2000). Upon cuprizone withdrawal after 6 weeks of administration, microglia numbers rapidly reduce, and this coincides with the peak of the remyelination phase (Mason et al., 2000). Interestingly, mice administered cuprizone for an extended time of 12 weeks exhibit chronic demyelination and maintain increased microglia numbers at sites of demyelination. Chronic cuprizone exposure also results in impaired remyelination capacity, though it is unknown if this effect is linked to the prolonged presence of microglia (Mason et al., 2004).

A recent scRNAseq study by Masuda et al. (2019) comparing control, cuprizone-demyelinated and cuprizone-remyelinated corpus callosum tissues provided a comprehensive picture of the diverse microglia populations at the various stages of damage and repair. Nine microglia clusters were identified in the naïve corpus callosum, with the prevalence of homeostatic clusters. Demyelination and remyelination states were associated with the overwhelming presence of two disease-associated clusters with inverse distribution. During demyelination, the most abundant cluster expressed inflammatory genes, many of which identical to those found in microglia of MS patients (e.g., osteopontin, PADI2, ApoE). During remyelination, the most abundant subset displayed an immunoregulatory gene signature. The clear similarity observed with disease-related subtypes found in the MS brain further validates the usefulness of the cuprizone model to study myelin repair mechanisms relevant to MS.

### Detrimental Microglial Responses in Cuprizone-Induced Demyelination

**Figure 2:**
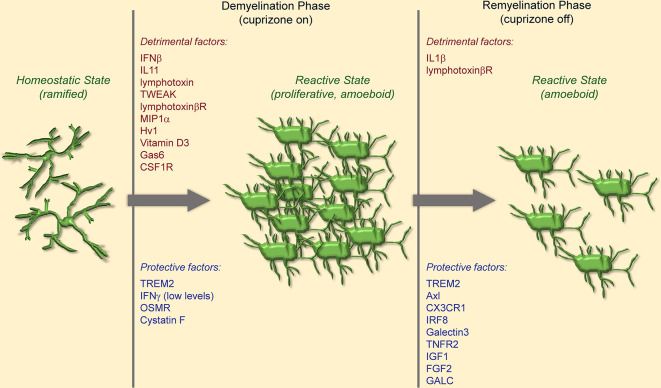
Schematic of microglia activation and responses in the cuprizone model of demyelination. Microglia become proliferative and reactive upon cuprizone administration and populate sites of demyelination. During demyelination, microglia are primarily detrimental through production of chemokines and cytokines that exacerbate inflammation and demyelination. During remyelination, microglia are primarily protective and express genes involved in debris phagocytosis, clearing the way for reparative remyelination.

Microglia are recruited to sites of demyelination in the cuprizone model and contribute to the inflammatory environment. Recruitment depends on chemoattractants such as CCL2, CCL3, and CXCL10, which are upregulated in the corpus callosum within 2 days of cuprizone administration. Ablation of any of these molecules leads to reduced microglia numbers in the corpus callosum and correlates with reduced and/or delayed demyelination (McMahon et al., 2001; Clarner et al., 2015; Janssen et al., 2016). Microglia accumulation in the demyelinated brain coincides with further upregulation of chemokines and cytokines (Morell et al., 1998; Jurevics et al., 2002; Arnett et al., 2003), resulting in an inflammatory environment that propagates microglia proliferation and activation (Figure 2).

Several studies suggest that activated microglia are responsible for oligodendrocyte death, not cuprizone acting directly on oligodendrocytes. Indeed, cuprizone administration to primary rat oligodendrocytes *in vitro* does not affect their viability even at high doses, nor does the concomitant addition of astrocyte conditioned media (Pasquini et al., 2007). Instead, the addition of microglia produced cytokines, such as TNF, reduced oligodendrocyte viability, suggesting that synthesis of inflammatory mediators by microglia is necessary for oligodendrocyte death and demyelination (Pasquini et al., 2007). However, TNF does not seem to be involved in the proliferation and recruitment of inflammatory microglia at the site of demyelination, as mice with TNF ablation do not show a reduction in microglia numbers or activation state following cuprizone administration (Arnett et al., 2001). On the contrary, microglia-derived TNF may be important for the remyelination process *via* stimulation of TNFR2 signaling in OPCs (reviewed below; Arnett et al., 2001).

Inhibition of microglial activation with minocycline in mice undergoing cuprizone treatment reduced demyelination, highlighting the detrimental function of microglia in this model (Skripuletz et al., 2010). Along this line, depletion of microglia by the administration of the CSF1R inhibitor PLX3397 during the remyelination phase resulted in increased remyelination rate and improved recovery of motor deficits (Tahmasebi et al., 2019). Short term treatment with the CSF1R kinase inhibitor BLZ945 also led to reduced microglia numbers which correlated with less demyelination and higher oligodendrocyte numbers in the corpus callosum. Additionally, the few remaining microglia had increased phagocytic and debris clearing ability (Wies Mancini et al., 2019). Targeting of microglial CD38, which, similarly to CSF1R, is important for microglial survival and homeostasis, has also been shown to reduce microglia numbers and activation after cuprizone exposure, protecting mice against demyelination and neurodegeneration (Roboon et al., 2019).

Production of ROS by microglia has been suggested as another mechanism driving oligodendrocyte death and demyelination. Indeed, mice lacking Hv1, a microglia specific voltage-gated proton channel required for ROS production in the brain, showed reduced demyelination and decreased microglia activation (Liu et al., 2015). On the other hand, conditional ablation of the antioxidant enzyme methionine sulfoxide reductase A (MsrA) in microglia resulted in increased production of ROS, decreased activity of SODs, and exacerbated demyelination (Fan et al., 2020).

Activation of signaling cascades favoring the switch of microglia towards a proinflammatory, proliferative phenotype has been correlated with increased demyelination and delayed remyelination. This is the case for IFNγ and IFNβ signaling. IFNγ promotes microglia recruitment to the demyelination site (Maña et al., 2006) and tolerance to IFNγ through constitutive expression of very low levels of IFNγ protects against demyelination (Gao et al., 2000). IFNβ has a detrimental effect as well, demonstrated by the fact that its ablation leads to reduced microglia inflammatory activation and accelerated remyelination (Trebst et al., 2007). TLR2 signaling plays a similar role. Its ablation resulted in enhanced remyelination (Esser et al., 2018), and so did TLR2 tolerance induced *via* administration of TLR ligands (Wasko et al., 2019). This was associated with a shift in phenotype from iNOS^+^ detrimental microglia to Arg1^+^ reparative microglia (Esser et al., 2018; Wasko et al., 2019). Other signals implicated in the detrimental activation of microglia are members of the TNF family of cytokines lymphotoxin (LT) and TWEAK. Indeed, inhibition of the LT beta receptor and suppression of TWEAK are both protective in cuprizone-induced demyelination (Plant et al., 2007; Iocca et al., 2008). Finally, miR-146a, which is upregulated following cuprizone administration, has been associated with microglia proliferation in the acute phase of cuprizone demyelination. Its ablation reduces microglia numbers and protects against demyelination of the corpus callosum (Martin et al., 2018). However, in a report by Zhang et al. (2017), continuous infusion of miR-146a mimics promoted remyelination in the corpus callosum, likely by suppressing inflammatory microglial activation, thus highlighting a difference in the function of miR146a during the demyelination vs. remyelination process.

At the opposite end, activation of signaling cascades leading to suppression of microglia inflammatory and proliferative phenotype is protective. One example is the activation of the kinase receptor Axl by its ligand Gas6. Mice ablated of these molecules exhibited increased axonal damage, decreased remyelination, and increased expression of proinflammatory cytokines after cuprizone exposure, indicating a suppressive role for the Axl-Gas6 axis (Ray et al., 2017). Protection against demyelination was observed with overexpression of the cytokine IL13, which promoted the polarization of microglia towards a suppressive Arg1^+^ phenotype (Guglielmetti et al., 2016).

As expected, based on these reports, pharmacological interventions leading to suppression of microglial inflammatory activation and recruitment have shown protection in the cuprizone model. The estrogen 17beta-estradiol (E2) delayed microglial recruitment and reduced gene expression of TNF limiting demyelination (Taylor et al., 2010). Lactacystin, a naturally occurring proteasome inhibitor, impaired microglia recruitment, and improved remyelination when injected into the corpus callosum (Millet et al., 2009), similar to the p53 inhibitor pifithrin alpha (Li et al., 2008). The antipsychotic drug olanzapine reduced both microglia accumulation and oligodendrocyte loss in the frontal cortex, which correlated with an increase in the protective growth factor IGF1 (Zhang H. et al., 2014). The phosphodiesterase 5 (PDE5) blocker sildenafil increased myelin preservation and decreased microglia numbers as well as microglia produced inflammatory cytokines (Nunes et al., 2012, 2016). Administration of vitamin D3 reduced demyelination and this correlated with reduced microglia numbers (Wergeland et al., 2011). In a separate study, vitamin D3 given during the sixth week of cuprizone administration increased microglia activation and exacerbated demyelination, but in the long run, increased remyelination and decreased microglial activation, suggesting that temporal control of microglia activity is essential to regulate demyelination/remyelination (Nystad et al., 2014). Pharmacological inhibition of 5-lipoxygenase (5-LO), which blocks the synthesis of inflammatory leukotrienes, reduced microglia activation and axonal damage (Yoshikawa et al., 2011), but did not prevent or limited demyelination, suggesting that microglia may participate in cuprizone-induced damage also by compromising neuronal integrity, not only oligodendrocyte integrity (Yoshikawa et al., 2011). Sulfasalazine, commonly used in rheumatoid arthritis, promotes repair of demyelinated lesions in cuprizone mice by preventing microglia from acquiring a proinflammatory profile, thus reducing their production of TNF and INFγ (Duan et al., 2018).

### Protective Microglial Responses in Cuprizone-Induced Demyelination

One of the key functions of microglia in physiological and pathological conditions is phagocytosis. Oligodendrocyte cell death associated with cuprizone administration results in the accumulation of myelin debris that microglia need to clear for proper remyelination to take place. Microglia phagocytic function is regulated by specific molecules, such as the surface receptor Triggering Receptor Expressed on Myeloid cells 2 (TREM2), which is elevated during demyelination (Konishi and Kiyama, 2018). TREM2 ablation compromises microglia phagocytic capacity, resulting in impaired myelin debris clearance and persistent demyelination (Cantoni et al., 2015; Poliani et al., 2015). Additionally, it has been shown that Galectin 3 is an important signal for TREM2 regulation during demyelination. Galectin 3 KO mice fail to upregulate TREM2 leading to a lack of spontaneous remyelination after cuprizone administration (Pasquini et al., 2011). Galactocerebrosidase (GALC), which has been identified as a risk factor for MS, also participates in microglia phagocytic clearance of myelin debris, with GALC ablated mice showing reduced remyelination (Scott-Hewitt et al., 2017). A similar phenotype was observed in mice lacking Axl (TAM receptor), CX3CR1, and interferon regulatory factor 8 (IRF8), all genes important for microglia homeostatic function. Their ablation leads to impaired myelin clearance and delayed recovery from cuprizone demyelination (Weinger et al., 2011; Horiuchi et al., 2012; Lampron et al., 2015).

In addition to phagocytosis, the immunomodulatory function of microglia is necessary for repair in cuprizone demyelination. Using knockout mice lacking TNF and its receptors, Arnett et al. showed that TNF, which is produced primarily by microglia, is necessary for remyelination by promoting OPC differentiation *via* activation of TNFR2 signaling (Arnett et al., 2001). Additionally, mice lacking MHCII, which is exclusively expressed by microglia in the intact CNS, exhibited delayed remyelination (Arnett et al., 2003). MHCII expression on microglia is regulated by TNF, whose ablation leads to improper microglia function and consequently reduced remyelination after cuprizone exposure (Arnett et al., 2001, 2003).

Production of protective/reparative soluble factors by microglia is beneficial in cuprizone demyelination. Cystatin F, a cathepsin inhibitor synthesized by microglia, is important for remyelination. Ablation of cystatin F increases demyelination and expression of CXCL2 after cuprizone exposure. This is reversed with cathepsin C gene knockdown, suggesting that microglial cystatin F protective effect is through inhibition of cathepsin C (Liang et al., 2016). Microglia derived CNTF promotes remyelination. Indeed, suppression of CNTF production with minocycline, which inhibits microglial activation, resulted in reduced remyelination (Tanaka et al., 2013).

Strategies that promote the polarization of microglia towards a reparative phenotype are protective in cuprizone demyelination. Treatment with the IL6 family member oncostatin M (OSM) shifted microglia to an anti-inflammatory phenotype and prevented demyelination, while a deficiency in the OSM receptor (expressed on microglia and astrocytes) exacerbated demyelination in the cuprizone model (Janssens et al., 2015). Progesterone treatment during cuprizone exposure reduced demyelination by shifting microglia from a pro-inflammatory to an anti-inflammatory phenotype with elevated expression of TREM2, CD206, Arg1, and TGFβ.

## Lysolecithin-Induced Demyelination

**Figure 3:**
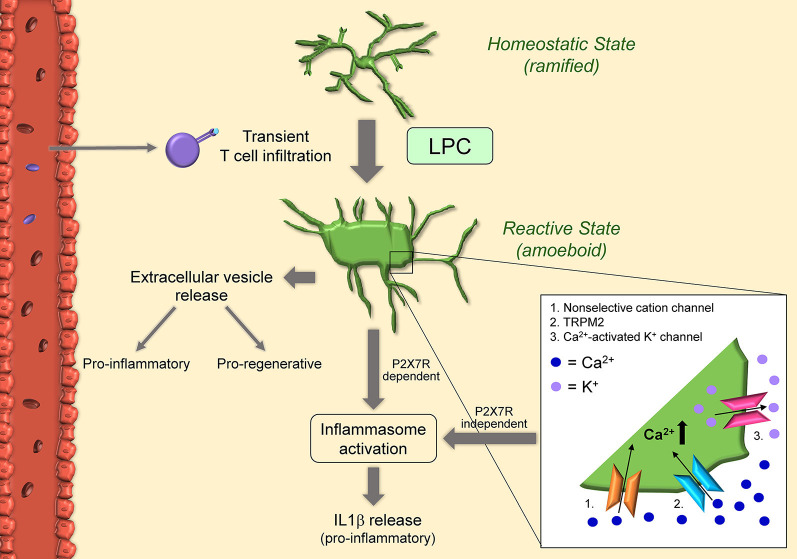
Schematic of microglial activation in the lysolecithin (LPC) model. Microglia become activated after exposure to LPC itself, and in response to T cells transiently present in the LPC-exposed CNS tissue. Once activated, microglia acquire amoeboid morphology via concurrent activation of non-selective cation channels (1) and KCl cotransporters: non-selective cation channels in the cell body increase osmolarity and cause swelling, while KCl cotransporters in the cell processes reduce osmolarity and cause shrinkage. LPC can also increase intracellular calcium by non-selective transient receptor potential melastatin 2 (TRPM2) (2). Calcium influx in turn activates microglia and calcium-activated K+ channels (3). This results in the release of pro-inflammatory IL1beta in a mechanism independent of P2X7R. Microglia can also release IL1beta via inflammasome activation. Indeed, LPC acts as a DAMP and results in activation of NLRP3 and NLRC4. Activated microglia also release extracellular vesicles with proinflammatory functions early after injection and pro-regenerative effects 7-10 days after injury.

Lysolecithin, or lysophosphatidylcholine (LPC), is a demyelinating and inflammatory phospholipid that acts as a membrane-dissolving detergent. When delivered by injection into white matter tracts, LPC induces highly reproducible focal demyelinating lesions. Injections are typically targeted to the thoracic and lumbar regions of the spinal cord or the corpus callosum (Blakemore and Franklin, 2008). Demyelination occurs within hours and can last up to 7–10 days, with noticeable remyelination observed by day 21. The insult causes peripheral macrophages to infiltrate into the lesion, as well as microglia to be recruited (Figure 3). Infiltration of T cells, although transient (between 6–12 h after injection), appears to be an important step in the activation of macrophages and microglia. Indeed, nude mice lacking a T cell response show marked reduction in phagocytic macrophages, activated microglia, and extent of demyelination after LPC injection (Ghasemlou et al., 2007). The involvement of peripheral immune cells in the development of LPC-induced demyelination differentiates this model from both the cuprizone model, where immune cells are not required for demyelination to occur, and the EAE model, where demyelination is strictly dependent on the induction of a primary T cell response. Advantages of the LPC model are the rapid establishment of demyelination lesions (hours as opposed to weeks in the cuprizone model), and the flexibility in choosing the lesion location.

A recent bulk RNAseq study of microglia isolated from LPC lesions during the demyelinating (3 days post-injury) and remyelinating phase (10 days post-injury), showed that in the demyelinating phase microglia have a predominantly proinflammatory transcriptome, typically associated with damaging functions, while in the remyelination phase microglia display a reparative/pro-regenerative profile. Interestingly, the necroptotic death of proinflammatory microglia is necessary for the replenishment of the cell pool with regenerative microglia which drives the remyelination process (Lloyd et al., 2019). ScRNAseq analysis at 7 days post-injury, when the lesion is transitioning from a state of myelin debris removal to the remyelination phase, revealed the existence of four distinct injury-associated microglia clusters, all of which with downregulated homeostatic genes (e.g., Cx3cr1, P2ry12) and upregulated inflammatory genes (e.g., Apoe, Cxcl10, Ccl2, Il1β, interferon pathway genes). These signatures were similar to those observed in human MS lesions and in other neurological diseases (e.g., Alzheimer’s; Hammond et al., 2019).

### Detrimental Microglial Responses in LPC-Induced Demyelination

*in vitro* studies have determined that microglia viability/integrity is not affected by LPC, differently from oligodendrocytes (Vereyken et al., 2009). Instead, LPC appears to influence the activation state of microglia. LPC exposure promotes inflammatory microglia activation and accumulation, and this response correlates with acute axonal damage (Höflich et al., 2016). Following LPC injection *in vivo*, as well as exposure *in vitro*, microglia transition from a steady-state ramified to an amoeboid activated morphology (Schilling et al., 2004; Stock et al., 2006; Jeong et al., 2017). Patch-clamp experiments have shown that LPC activates non-selective cation channels and KCl cotransporters (Schilling et al., 2004), the first highly expressed in the cell body, and the latter mostly expressed in the processes. The hypothesis is that influx of ions through non-selective cation channels increases osmolarity in the cell body resulting in swelling. In parallel, KCl cotransporters reduce osmolarity in the processes, causing shrinkage and retraction. The synergy of these two mechanisms directs microglia towards an activated amoeboid morphology (Schilling et al., 2004). Furthermore, non-selective cation channels and calcium-activated potassium channels seem to be required for processing and release of the inflammatory cytokine IL1β from LPS-preactivated microglia after exposure to LPC with a mechanism independent of P2RX7 activation (Stock et al., 2006). More recent studies have demonstrated that LPC acts essentially as a danger-associated molecular pattern (DAMP) and induces IL1β release by microglia *in vitro* *via* both NLRP3 and NLRC4 inflammasomes through the canonical inflammasome pathway (Freeman et al., 2017; Scholz and Eder, 2017). Both inflammasomes are important signals in the innate immune response to pathogens. Specifically, LPS-preactivated microglia from NLRP3 and NLRC4 knockout mice showed a significant reduction in IL1β release after LPC stimulation, suggesting both NLRP3 and NLRC4 inflammasomes contribute to caspase-1 activation for IL1β release to occur (Freeman et al., 2017). This indicates that LPC can act both extracellularly and intracellularly. Indeed, by binding to membrane receptors, LPC may activate mechanosensitive non-selective cation channels, which could trigger the activation of the NLRP3 inflammasome (Scholz and Eder, 2017). Also, LPC could cross the cell membrane due to its lipophilic nature and interact intracellularly with the NLRC4 inflammasome (Scholz and Eder, 2017). It has also been shown that LPC can potentiate P2X7R-mediated responses, such as the formation of membrane pores, activation of p44/42 MAPK, and calcium influx (Takenouchi et al., 2007). This may be due to LPC increasing the sensitivity of microglial P2X7R in the brain. LPC-dependent modulation (increase) of intracellular calcium and, consequently, increase in microglial activation has also been linked to non-selective transient receptor potential (TRP) channels, specifically TRPM2, *via* activation of p38 MAPK signaling (Jeong et al., 2017).

Much of the detrimental functions of microglia in the LPC model have been attributed to the inflammatory microglia pool present during the demyelination phase. This pool shows upregulation of genes associated with chronic inflammation and cell-death mechanisms (Lloyd et al., 2019). It has been suggested that damaging microglia carry out their function through the shedding of extracellular vesicles (EVs) loaded with signals (RNA and proteins) that cause oligodendrocyte cell death, prevent OPC differentiation and inhibit remyelination (Lombardi et al., 2019).

### Protective Microglial Responses in LPC-Induced Demyelination

Microglia phagocytic function has been shown to have a protective debris clearing role in LPC-induced demyelination, similar to both the EAE and cuprizone models. An important signal for the phagocytic activity of myeloid cells in LPC lesions is miR-223. Ablation of miR-223 resulted in larger lesions and impaired remyelination with the accumulation of lipid-laden microglia/macrophages unable to process myelin debris. Not only did microglia fail to clear myelin, but became more inflammatory (Galloway et al., 2019a).

Aside from phagocytosis, the reparative roles of microglia in the LPC model have been mainly associated with the remyelination phase. At this stage, microglia return to expressing homeostatic genes (e.g., Csfr1 and P2ry12) and upregulate genes associated with oligodendrocyte differentiation and myelin formation (e.g., Osm, Fgf1, Bmp1; Lloyd et al., 2019). A recent study demonstrated that the reparative effects of pro-regenerative microglia are associated with the extracellular vesicles (EVs) they shed. These EVs carry signals that promote OPC recruitment and differentiation at LPC-induced lesions favoring remyelination (Lombardi et al., 2019).

## Concluding Remarks

Microglia play a key role in MS. Their cellular diversity as well as complex functions in the pathophysiology of the disease are increasingly being appreciated. Microglia’s multifaceted and often dichotomous responses vary upon the stage of disease (onset, acute, chronic) and their anatomical location in the brain and spinal cord (e.g., within lesions, in NAWM, in gray matter). While detrimental functions are highly associated with MS onset and acute disease, protective functions have been attributed to microglia in facilitating the repair process. Thus, to be successful in uncovering the underlying pathological mechanisms of MS and, consequently, identifying novel drug targets, it is paramount that animal models to study MS accurately reflect the complexity and diversity of microglia responses. What emerges from the numerous studies in the EAE, cuprizone, and LPC models is that microglia indeed display signatures and responses highly representative of the various stages of lesion formation and resolution in the human disease. Generally speaking, in all models detrimental microglial activation is maximal at the acute phase of the disease (meaning peak EAE, and peak demyelination in cuprizone and LPC), whereas microglia-dependent reparative functions best correlate with sub-acute and chronic EAE, or the remyelination phase in cuprizone and LPC models. While T cell involvement is significant in the EAE model, and present to some extent in the LPC model, with cuprizone administration T cells are of minimal to no consequence. This makes the cuprizone model the “cleanest” to appreciate microglial-dependent innate immune mechanisms and to explore avenues to directly affect oligodendrocyte survival and differentiation and promote remyelination. In summary, this overview of the breadth of microglial responses provides validation of the usefulness of the models commonly utilized to recapitulate the different aspects of MS immunopathology, thus a degree of confidence that findings with these tools may be translated to MS therapy.

## Author Contributions

MP and HD equally contributed to drafting the manuscript and the figures. RB identified the topic, drafted the manuscript and the figures. All authors contributed to the article and approved the submitted version.

## Conflict of Interest

The authors declare that the research was conducted in the absence of any commercial or financial relationships that could be construed as a potential conflict of interest.

## Abbreviations

5-LO: 5-lipoxygenase
AMPK: AMP-activated protein kinase
APC: Antigen presenting cells
A*RG*1: Arginase 1
ASC: Apoptosis-associated speck-like protein
ATP: Adenosine triphosphate
BBB: Blood-brain barrier
Ca: Calcium
CD11b: Cluster of differentiation molecule 11b
CD206: Mannose receptor
CD3: cluster of differentiation 3
CD38: cluster of differentiation 38
CD40: cluster of differentiation 40
CD45: cluster of differentiation 45
CD68: cluster of differentiation 68
CNS: Central nervous system
CNTF: Ciliary neurotrophic factor
CSF: Cerebro spinal fluid
CSF1: Colony-stimulating factor 1
CX3CR1: CX3C chemokine receptor 1
CXCR4: CXC chemokine receptor type 4
daMG: Damage associated microglia
DAMP: Damage associated molecular pattern
EAE: Experimental autoimmune encephalomyelitis
ERβ: Estrogen receptor β
ErbB-1: Epidermal growth factor receptor 1
EV: Extracellular vesicles
FLT-1: Vascular endothelial growth factor receptor 1
Gal-1: Galactokinase
GALC: Galactocerebrosidase
GAS6: Growth arrest specific 6
GM: Gray matter
HLA-DR: Human leukocyte antigen-DR isotype
Hv1: Voltage gated hydrogen channel 1
IFN: Interferons
IFNAR1: Interferon alpha and beta receptor subunit 1
IGF1: Insulin growth factor 1
iNOS: Inducible nitric oxide synthase
I.P.: Intraperitoneal
IRAK: Interleukin-1 receptor associated kinase 1
IRF8: Interferon regulatory factor 8
KO: Knock-out
LPC: Lysophosphatidylcholine/Lysolecithin
MAPK: Mitogen-associated protein kinase
MBP: Myelin basic protein
MD1: Lymphocyte antigen 86
MHC: Major histocompatibility complex
MOG: Myelin oligodendrocyte glycoprotein
MP: Methylprednisolone
MPO: Myeloperoxidase
MS: Multiple sclerosis
MSRA: Methionine sulfoxide reductase A
NADPH: Nicotinamide adenine dinucleotide phosphate
NAGM: Normal appearing gray matter
NAWM: Normal appearing white matter
NF-κB: Nuclear factor kappa-light-chain-enhancer of activated B cells
NLRC4: NLR family CARD domain containing 4
NLRP3: NLR family Pyrin domain containing 3
NO: Nitric oxide
NOD: Non-obese Diabetic mouse strain
NOX2: NADPH oxidase 2
OPC: Oligodendrocyte precursor cells
OSM: Oncostatin M
P2RX7: Purinergic receptor P2X7
P2RY12: Purinergic receptor P2Y12
P2X4R: Purinergic receptor P2X4
PET: Positron emission tomography
PLP: Proteolipid protein
ROS: Reactive oxygen species
RRMS: Relapsing-remitting multiple sclerosis
S.C.: Subcutaneous
SOD: Superoxide dismutase
TAK1: Mitogen activated protein kinase kinase kinase 7
TAM: Tyro3, Axl and MerTK receptors
TGFβ1: Transforming growth factor beta 1
TLRs: Toll-like receptors
TMEM119: Transmembrane protein 119
TNF: Tumor necrosis factor
TNFR: Tumor necrosis factor receptors
TRAF3: TNF receptor-associated factor 3
TREM2: Triggered receptor expressed on myeloid cells 2
TRPM2: Transient receptor potential cation channel subfamily M member 2
TSPO: Translocator protein
TWEAK: Tumor necrosis factor-like weak inducer of apoptosis
VEGF-B: Vascular endothelial growth factor B
WM: White matter
WT: Wild type.
